# Participation inequality in the National General Health Examination based on enterprise size

**DOI:** 10.1186/s40557-017-0159-y

**Published:** 2017-02-22

**Authors:** Young Joong Kang, Jong Heun Park, Huisu Eom, Bohwa Choi, Seyoung Lee, Ji-Won Lee, Jun-Pyo Myong

**Affiliations:** 1 Occupational Safety and Health Research Institute, Korea Occupational Safety and Health Agency, 400, Jongga-ro, Jung-gu Ulsan, Republic of Korea; 2 Big Data Steering Department, National Health Insurance Service, 32, Sambo-ro, Wonju-si, Gangwon-do Republic of Korea; 3 Department of Occupational and Environmental Medicine, Seoul St. Mary’s Hospital, College of Medicine, Catholic University of Korea, 222 Banpo-Daero Seocho-gu, Seoul, 06591 Republic of Korea

**Keywords:** Mass screening, Healthcare disparities, Size, Enterprise, Policyholder

## Abstract

**Background:**

Health examinations are performed so that diseases can be identified and treated earlier. Several studies have evaluated the determinants of participation in health examinations including cancer screening, but few have evaluated the relationship between the size of the enterprise and their participation in Workers’ General Health Examinations (WGHE). The aim of the present study was to estimate the association of WGHE participation with the size of the enterprise and the type of policyholder.

**Methods:**

The eligible population from 2006 through 2013 was extracted from the National Health Insurance Service (NHIS) database. The population size ranged from 14–17 million. After adjustment for age and gender, multiple logistic regression analysis was performed to estimate the odds ratios of participating in the WGHE (by age group) based on the type of policyholder (reference: public officers) and the size of the enterprise (reference: enterprise size ≥300 employees), respectively.

**Results:**

Workers employed at enterprises with <50 persons were less likely to participate in WGHEs than those employed at enterprises with ≥300 persons. After policyholders were stratified by type (non-office workers vs. public officers), a disparity in the WGHE participation rate was found between the different types of policyholders at enterprises with <50 employees (reference: those employed at enterprises with ≥300 employees); the odds ratios for subjects in their 40s and 50s were 0.2–0.3 for non-office workers vs. 0.8–2.0 for public officers.

**Conclusion:**

Workplace policyholders at small enterprises comprised a vulnerable group less likely to participate in WGHEs. Efforts should be made to raise the WGHE participation rate among the vulnerable employees belonging to small enterprises, as well as among their dependents.

## Background

Health examinations are performed so that diseases can be identified and treated earlier. Health examinations are classified as either organized or opportunistic. Health examinations for workers have been recognized under the Labor Standards Act in Korea since 1953. After 1972, health examinations for workers were separated into Workers’ Specific Health Examination (WSHE) and Workers’ General Health Examinations (WGHE). The National Health Insurance Service (NHIS), which began managing health insurance and health examinations in 1995, provides the National General Health Examination (NGHE) for workers, also known as WGHE. WGHEs are now provided every year or every other year, depending on the type of policyholder [1, 2].

Several studies have evaluated the associations of those determinants with participation in health examinations [3–7]. The determinants of participation in health examinations vary, and can be classified into predisposing factors, such as age, gender, predisposing diseases, concerns, etc., and possible factors such as income, education, self-reported socioeconomic status, occupation, etc. Regarding working conditions, previous studies have classified the type of work using binary (employed or unemployed) [4] or tertiary divisions (manual, non-manual, and unemployed) [3, 5].

From the perspective of occupational health, the size of enterprise affects the vulnerability of the individual in the work environment. Because the size of an enterprise may influence the health outcomes of the employees by affecting the quality of the working environment, the time available for health examinations, or their job stability [8]. However, few studies have evaluated the relationship between enterprise size and WGHE participation.

The aim of the present study was to estimate the associations of policyholder type and enterprise size with WGHE participation using the NHIS database. In addition, we evaluated the odds of participating in a WGHE based on the size of the enterprise after stratifying subjects in their 40s and 50s by policyholder type.

## Methods

### Data source and study population

The present study dataset was derived from the NHIS. The NHIS dataset consists of four databases (DBs): qualification, medical treatment, WGHE, and medical institution. The dataset for this study was derived from the qualification and health examination DBs. The information about age, gender, type of policyholder, and enterprise size was extracted from the qualification DB, and information about participation was taken from the WGHE DB. According to the Occupational Safety and Health Acts, the WGHE for an Workplace Policyholder (WP) should be done for office workers and public officers every second year, and for non-office workers annually. According to the Law for Health Promotion, dependents or household members (40 years and above) of an WP or a Regional Policyholder (RP) should receive an examination every other year. Medical Aid Beneficiaries must undergo biannual health examinations [1, 2]. The eligible population from 2006 through 2013 was extracted based on the criteria described above. The population ranged 14–17 million (Table 1).

**Table 1:** General characteristics of eligible population by year

	2006		2007		2008		2009		2010		2011		2012		2013	
	N	%	N	%	N	%	N	%	N	%	N	%	N	%	N	%
Age	47.2	14.2	47.8	14.4	47.5	14.3	48.3	14.4	48.6	14.3	48.6	14.4	49.1	14.4	49.7	14.4
Gender																
Male	8,345,124	55.4	7,856,987	54.7	8,992,965	54.5	8,762,242	53.8	9,201,725	54.0	8,814,474	54.0	8,985,416	53.7	9,049,455	53.5
Female	6,708,637	44.6	6,501,722	45.3	7,500,836	45.5	7,514,997	46.2	7,838,049	46.0	7,518,990	46.0	7,745,624	46.3	7,862,009	46.5
Type																
RPs & dependents	7,991,124	53.1	7,907,606	55.1	8,182,204	49.6	8,411,953	51.7	8,643,198	50.7	8,046,862	49.3	8,074,084	48.3	8,178,034	48.4
Office worker	1,515,523	10.1	1,072,442	7.5	2,782,176	16.9	2,732,174	16.8	2,816,343	16.5	3,061,514	18.7	2,975,592	17.8	3,042,002	18.0
Non-office worker	4,623,043	30.7	5,100,114	35.5	4,629,334	28.1	4,892,934	30.1	4,890,706	28.7	4,928,176	30.2	5,043,341	30.1	5,366,346	31.7
Public officers	924,071	6.1	278,547	1.9	900,087	5.5	240,178	1.5	689,527	4.1	296,912	1.8	638,023	3.8	325,082	1.9
Participation																
No	6,645,543	44.2	5,939,177	41.4	6,229,381	37.8	5,739,373	35.3	5,548,044	32.6	4,593,690	28.1	4,647,764	27.8	4,802,579	28.4
Yes	8,408,218	55.9	8,419,532	58.6	10,264,420	62.2	10,537,866	64.7	11,491,730	67.4	11,739,774	71.9	12,083,276	72.2	12,108,885	71.6
Total	15,053,761		14,358,709		16,493,801		16,277,239		17,039,774		16,333,464		16,731,040		16,911,464	

For Age, means and standard deviation

*RP* Regional policyholder

### Definition of policyholder and enterprise size

WPs were classified into office workers, non-office workers, and public officers. The detailed definitions are reported elsewhere [2]. The households or family members (who did not have incomes and were over 40 years old) belonging to the previously defined WPs and RPs were defined as dependents. Medical Aid was excluded from the present study [1]. The sizes of enterprises were classified as ≥300, 50–299, and <50 based on the number of regular employees.

### Statistical analysis

All statistical analyses were performed with SAS Enterprise 4.3 (SAS Institute, Cary, NC). After adjustment for age and gender, multiple logistic regression analysis was performed to estimate the odds ratios for participating in the WGHE (by age group) based on the type of policyholder (reference: public officer) or enterprise size (reference: enterprise size ≥300). Those two reference groups were relatively stable and guaranteed the participation of WGHE, therefore, the authors set those two groups were reference groups. Because people in their 40s and 50s are vulnerable to the lifestyle-related diseases targeted by the WGHE, we further analyzed selected results from the multiple logistic regression analysis among people in their 40s and 50s (by calendar years 2006–2013). An additional analysis of enterprise size based on the type of policyholder among WPs was also done.

## Results

The overall participation rate in the NGHE was 55.9% in 2006 and 71.6% in 2013 (Table 1). Over 50% of the eligible participants in the WGHE were WPs and public officers in 2013. The annual eligible population for the WGHE ranged from 14,358,709 in 2007 to 17,039,774 in 2010.

The NGHE participation rates by type of policyholder for the NHIS are shown in Table 2. The participation rate increased from 36% in 2006 to 59.6% in 2013 among RPs and dependents. For non-office workers, the participation rate ranged from 79.6% to 90.6%. Over 80% of public officers participated in the WGHE during the study years, except for 2007.

**Table 2:** Participation rate for National General Health Examination or Workers’ General Health Examination by type of policyholder

Calendar year	Participation	RP & dependents		Office worker		Non-office worker		Public officers	
		N	%	N	%	N	%	N	%
2006	No	5,110,899	64.0	437,274	28.9	945,129	20.4	152,241	16.5
	Yes	2,880,225	36.0	1,078,249	71.1	3,677,914	79.6	771,830	83.5
	Total	7,991,124		1,515,523		4,623,043		924,071	
2007	No	4,717,810	59.7	232,925	21.7	923,421	18.1	65,021	23.3
	Yes	3,189,796	40.3	839,517	78.3	4,176,693	81.9	213,526	76.7
	Total	7,907,606		1,072,442		5,100,114		278,547	
2008	No	4,651,095	56.8	738,024	26.5	720,013	15.6	120,249	13.4
	Yes	3,531,109	43.2	2,044,152	73.5	3,909,321	84.4	779,838	86.6
	Total	8,182,204		2,782,176		4,629,334		900,087	
2009	No	4,360,434	51.8	682,419	25.0	652,252	13.3	44,268	18.4
	Yes	4,051,519	48.2	2,049,755	75.0	4,240,682	86.7	195,910	81.6
	Total	8,411,953		2,732,174		4,892,934		240,178	
2010	No	4,172,755	48.3	728,312	25.9	563,799	11.5	83,178	12.1
	Yes	4,470,443	51.7	2,088,031	74.1	4,326,907	88.5	606,349	87.9
	Total	8,643,198		2,816,343		4,890,706		689,527	
2011	No	3,303,175	41.0	758,846	24.8	485,263	9.8	46,406	15.6
	Yes	4,743,687	59.0	2,302,668	75.2	4,442,913	90.2	250,506	84.4
	Total	8,046,862		3,061,514		4,928,176		296,912	
2012	No	3,138,510	38.9	855,579	28.8	584,841	11.6	68,834	10.8
	Yes	4,935,574	61.1	2,120,013	71.2	4,458,500	88.4	569,189	89.2
	Total	8,074,084		2,975,592		5,043,341		638,023	
2013	No	3,302,678	40.4	950,361	31.2	505,778	9.4	43,762	13.5
	Yes	4,875,356	59.6	2,091,641	68.8	4,860,568	90.6	281,320	86.5
	Total	8,178,034		3,042,002		5,366,346		325,082	

**Fig. 1:**
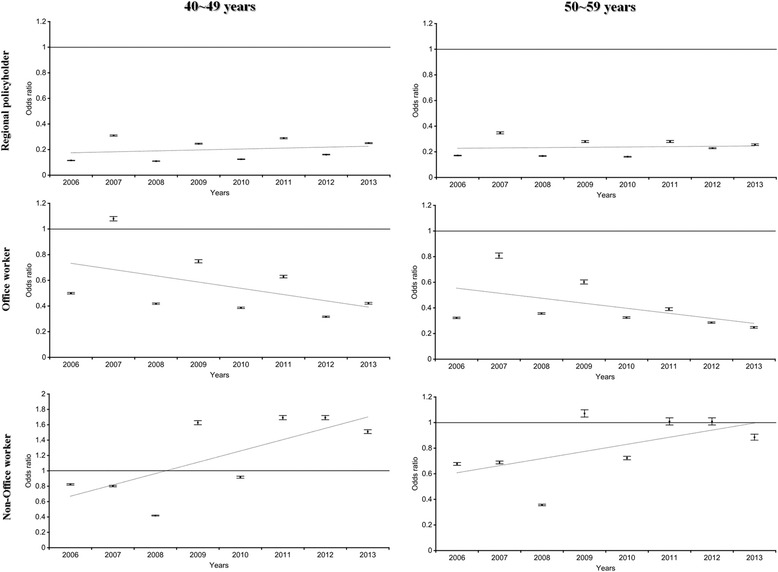
The odds ratio of type of policyholders (reference: public officer) for participating the national health examination

For the focus group of the WGHE (people in their 40s and 50s), the odds ratios for participating in the examination based on the type of policyholder (reference: public officers) are shown in Fig. 1. Regional policyholders and dependents and office workers in both age groups (40s and 50s) were less likely than public officers to participate in WGHEs. However, non-office workers were likely to participate.

Table 3 displays the distribution of enterprise sizes among WPs. The proportion of non-office workers were higher in those enterprise size ≥ 300 employees than in those enterprise size < 50 employees throughout the study period.

**Table 3:** Distribution of enterprise size by type of policyholders

Calendar year	Type of policyholders	Enterprise size (numbers of employees)					
		≥300		50 ~ 299		<50	
		N	%	N	%	N	%
2006							
	Office worker	492,568	18.7	333,665	18.3	689,288	26.4
	Non-office worker	1,614,653	61.4	1,182,628	64.9	1,825,714	69.9
	Public officers	521,331	19.8	307,195	16.8	95,545	3.7
2007							
	Office worker	323,546	14.3	219,518	14.0	529,353	20.2
	Non-office worker	1,766,269	78.0	1,260,830	80.5	2,072,641	79.1
	Public officers	175,712	7.8	84,940	5.4	17,891	0.7
2008							
	Office worker	600,424	21.5	554,446	27.3	1,627,274	46.6
	Non-office worker	1,688,128	60.6	1,178,849	58.1	1,762,302	50.4
	Public officers	498,855	17.9	295,195	14.6	106,030	3.0
2009							
	Office worker	540,225	21.5	469,143	25.8	1,722,795	48.6
	Non-office worker	1,835,440	73.2	1,258,913	69.3	1,798,574	50.8
	Public officers	131,215	5.2	88,935	4.9	20,028	0.6
2010							
	Office worker	717,635	24.7	478,472	23.5	1,620,066	46.9
	Non-office worker	1,889,278	65.1	1,275,242	62.6	1,725,948	49.9
	Public officers	294,943	10.2	284,148	13.9	110,436	3.2
2011							
	Office worker	636,648	23.2	482,367	25.5	1,942,349	53.2
	Non-office worker	1,943,642	70.9	1,300,853	68.8	1,683,254	46.1
	Public officers	162,790	5.9	106,966	5.7	27,154	0.7
2012							
	Office worker	767,567	24.8	524,600	24.8	1,683,406	48.8
	Non-office worker	2,041,165	66.0	1,341,340	63.4	1,660,739	48.2
	Public officers	283,646	9.2	251,347	11.9	103,030	3.0
2013							
	Office worker	621,256	20.9	456,864	22.6	1,963,746	52.5
	Non-office worker	2,168,763	73.0	1,454,696	71.9	1,742,681	46.6
	Public officers	180,793	6.1	112,257	5.5	31,969	0.9

**Fig. 2:**
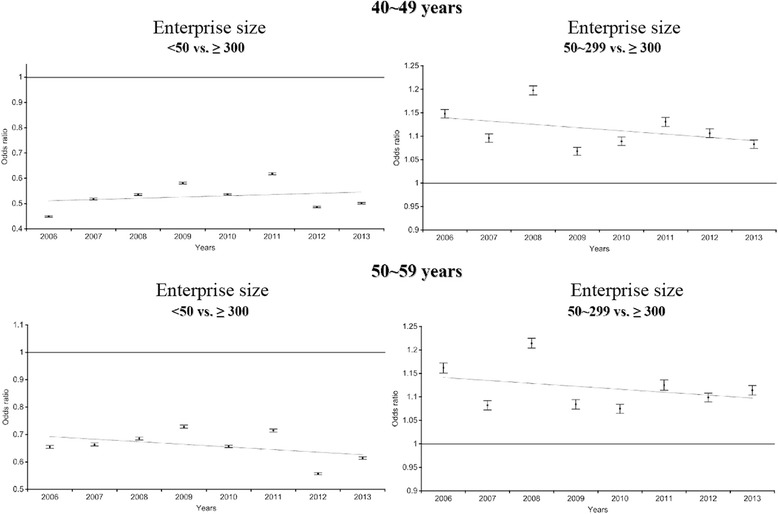
The odds ratio of company size (reference: ≥ 300) for participating the national health examination by 40s and 50s

The odds ratios of participating in the WGHE based on enterprise size (reference: enterprise size ≥300 employees) among subjects in their 40s and 50s during the eight study years are shown in Fig. 2. Workers at enterprises of <50 persons were less likely to participate in WGHEs than those employed at enterprises of ≥300 persons (odds ratios ranged from 0.47 [95% confidence interval (CI): 0.44–0.45] in 2006 to 0.62 [95% CI: 0.61–0.62] in 2011) among subjects in their 40s). The odds ratios for WGHE participation based on enterprise size (enterprise size <50 employees vs. ≥ 300 employees [reference]) ranged from 0.65 to 0.73 among subjects in their 50s. However, those employed at enterprises with 50–299 employees were more likely to participate in WGHEs than those employed at enterprises with ≥300 employees (*p <* 0.05).

**Fig. 3:**
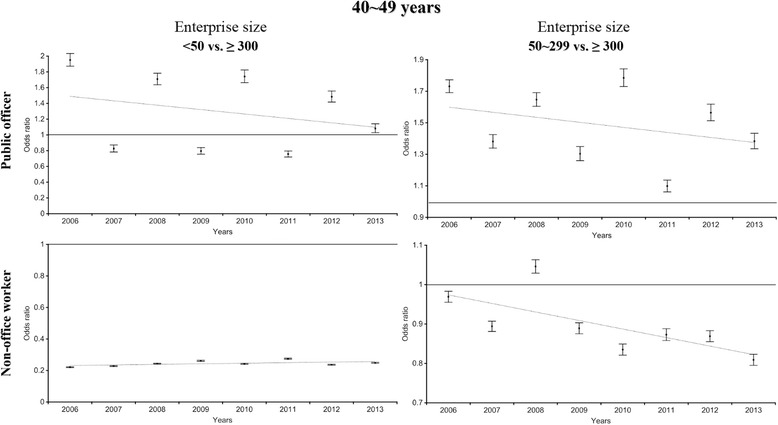
The odds ratio of company size (reference: ≥ 300) for participating the national health examination among 40s (stratified by public officer and non-office worker)

**Fig. 4:**
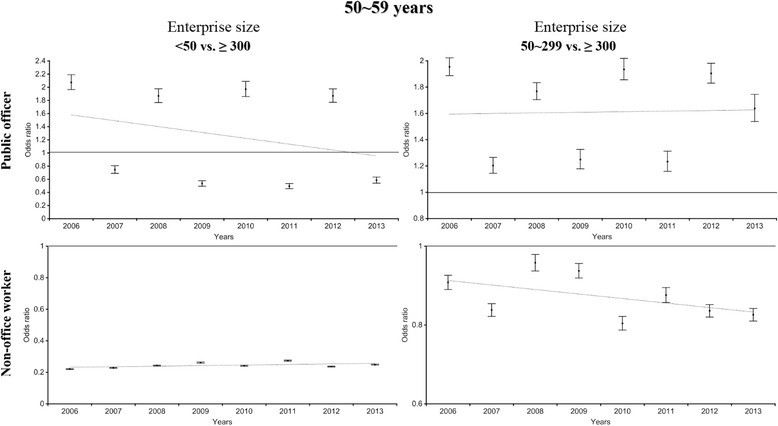
The odds ratio of company size (reference: ≥ 300) for participating the national health examination among 50s (stratified by public officer and non-office worker)

Stratified multiple logistic regression analysis was performed to estimate the inequality in WGHE participation based on enterprise size after stratification by type of policyholder (Figs. 3 and 4). After policyholders were stratified (non-office workers vs. public officers), a disparity in the WGHE participation rate was found between different types of policyholders employed at enterprises of <50 employees (reference: those employed at enterprises of ≥300 employees); the odds ratios for subjects in their 40s and 50s ranged from 0.2 to 0.3 among non-office workers vs. 0.8 to 2.0 among public officers. Likewise, for enterprises of 50–299, after stratification by type of policyholder, the odds ratio (reference: those employed at enterprises of ≥300 person) for participating in the WGHE among non-office workers was less than 1. Overall, the direction of the association by enterprise size differed between public officers and non-office workers.

## Discussion

In the present study, the participation rates in WGHEs by the NHIS were higher among WPs and public officers than among RPs and dependents. Enterprise size had different effects on WGHE participation for WPs and public officers. Employee insured workers at small enterprises comprised a vulnerable group less likely to participate in WGHEs.

There are several possible reasons for the difference in the NGHE participation rate between workers (including WPs and public officers) and RPs and their dependents. Since the beginning of the WGHE, the participation rate has been higher among WPs than among RPs and their dependents [9]. First, this is because the government imposes a fine on employees for not participating in the WGHE [1, 2], while there is no enforcement for RPs and dependents. Second, the accessibility of WGHE sites is another reason for the different participation rates; while some workers are able to participate in WGHEs at their workplaces, most of RPs and their dependents should participate in NGHEs at specific healthcare institutions. Third, the ages of policyholders and their dependents might affect the participation rate. With literatures concerning on the participation rate among RPs and dependents [10, 11], an age may be determinants of NGHE participation [12]. Although workers of all age ranges should participate in the WGHE, dependents 40 years of age or older were defined as targets of the WGHE. Fourth, according to the Occupational Safety and Health Acts, all workers should participate in WGHEs, and employers should give their employees paid vacation or early leave to encourage their participation. A self-employed person without any employees is classified as a RP. Such a person will lose income by participating in a NGHE. This is also a barrier to participating in NGHEs among RPs. Fifth, participation in an opportunistic health examination is another possible reason for lower participation in WGHEs among RPs and dependents. A previous study with 10,254 participants from the Korean Longitudinal Study of Ageing revealed the difference in participation in opportunistic health examinations between employed and unemployed individuals (odds ratio for the employed: 0.86 [95% CI: 0.75–1.00; ref: unemployed]) [4]. This reflects the fact that RPs and dependents have already used the opportunistic health examination. Therefore, the participation in an organized health examination like the NGHE might be lower among RPs. Those might result in the difference in the NGHE/WGHE participation rates among policyholders in the present study. To understand why those differences in the NGHE/WGHE participation rate, a more detail consideration on barriers for the NGHE/WGHE participation should be considered.

Enterprise size significantly impacts many aspects of workers’ lives, including their health [8, 13, 14]. The participation inequality for WGHEs is shown in Fig. 2 (enterprise size <50 employees vs. ≥300 employees). Historically, most small enterprises have been private companies, which might be fundamentally vulnerable [15]. The institutions administering the WGHE are unwilling to visit small enterprises due to the small number of employees who are eligible participants. Knowledge about and interest in health at small enterprises is poor [16]. Therefore, little effort has been made to improve poor workplace environments. In addition, due to the lower salary offerings, small enterprises are composed of vulnerable workers with regard to education, knowledge, physical status (unhealthy conditions), etc. At large enterprises, there is a greater guarantee of a stable labor environment (e.g., one with a labor union and welfare system) than at small enterprises [8]. With this knowledge, employees at small enterprises have been hesitant to participate in WGHEs.

As shown in Figs. 3 and 4, the WGHE participation inequalities became more prominent after the analysis was stratified by type of policyholder. Among employee insured non-office workers, those in large enterprises were previously shown to participate in the pre-employment health examination (now known as the pre-replacement health examination) than those in small enterprises (large enterprises: 89.4% vs. small enterprises: 30.4%) [17]. In addition, unhealthy workers might be forced to move to smaller enterprises, and under poor working conditions, they might lose the chance to participate in an organized health examination, even though it would be free [8, 18]. This phenomenon was also apparent in enterprises of 50–299 employees. On the other hand, WGHE participation inequalities were less likely among public officers at enterprises of <50 or 50–299 employees. The welfare system, including the health examinations of the national or local government, might support the participation of public officers in health examinations. Unstable employment conditions in Korea are less likely for public officers than for other employee insured policyholders.

Another reason for WGHE participation inequalities is the employee turnover rate at small enterprises in Korea. An issue paper evaluating the labor environment by enterprise size and type of employment revealed that the proportion of non-regular employees decreased as the enterprise size increased (the proportion of non-regular employees was 78.4% among enterprises of <50 employees vs. 14.3% among enterprises of ≥300 employees) in 2013 [19]. In addition, the authors demonstrated that the proportion of short-term employees (work duration ≤1 year) was higher among non-regular employees than among regular employees. The resignation rate was 0.48 for non-regular workers vs. 0.21 for regular workers in 2012 [15]. Thus, employee turnover within one year was likely at small enterprises [15]. At small enterprises, even non-office workers (who must undergo an annual WGHE) were likely to turn over in a year and lose the opportunity to participate in an annual WGHE. Therefore, efforts should be made to encourage employees at small enterprises to participate in WGHEs.

The present study had several limitations. First, policyholders were stratified into RPs and dependents, office workers, non-office workers, and public officers, but the eligible population among each category of policyholders was different in age. The enrollment criterion for dependents was being over 40 years old. Therefore, for those policyholders, the groups might have been heterogeneous. Secondly, other factors influence WGHE participation, such as the person’s socioeconomic status, position in the workplace, working duration, daily working hours, participation in shift work, marital status, but these risk factors were not included in the multiple logistic regression model. Although we searched for this information in the NHIS DB, it was not possible to access information about the work environment such as the working conditions. Nevertheless, the type of policyholder and the size of the enterprise might reflect the socioeconomic status. Third, the opportunistic health examination was not considered. Although the entire national database was assessed, it was not possible to estimate the participation in the opportunistic health examination. The opportunistic health examination should be further evaluated in future studies.

In spite of these limitations, this study also had several strengths. First, the national database powered by the NHIS was used to estimate the results. The NHIS covered about 90% of the Korean population in 2011 [20]. Therefore, our study subjects were likely to represent the population. Second, the association between enterprise size and WGHE participation was estimated after the data were stratified by the type of policyholder. As shown in Figs. 2 and 3, a negative association between the size of the enterprise and the type of policyholder was found in enterprises with 50–299 employees.

## Conclusion

The present study demonstrated that the NGHE/WGHE participation rates differed among different types of policyholders in the NHIS. Employee insured non-office workers at small enterprises comprised a vulnerable group less likely to participate in WGHEs. Although the General Health Examination (GHE) participation rate was higher among WPs than among RPs, WPs were also vulnerable to WGHE participation inequalities at small enterprises. The GHE is a fundamental right of NHIS policyholders. Efforts should be made to raise the WGHE participation rate in the vulnerable group of employees belonging to small enterprises, as well as among regional policyholders and dependents.

## Abbreviations

CI: Confidence interval
DBs: Databases
GHE: General Health Examination
NHIS: National Health Insurance Service
RP: Regional policyholder
WGHE: Workers’ General Health Examinations
WP: Workplace policyholder
WSHE: Workers’ Specific Health Examination
